# A Latent Class Analysis on Symptoms of Prolonged Grief, Post-Traumatic Stress, and Depression Following the Loss of a Loved One

**DOI:** 10.3389/fpsyt.2022.878773

**Published:** 2022-05-27

**Authors:** Carina Heeke, Minita Franzen, Hendrik Hofmann, Christine Knaevelsrud, Lonneke I. M. Lenferink

**Affiliations:** ^1^Department of Clinical-Psychological Intervention, Freie Universität Berlin, Berlin, Germany; ^2^Department of Clinical Psychology and Experimental Psychopathology, Faculty of Behavioral and Social Sciences, University of Groningen, Groningen, Netherlands; ^3^Department of Psychology, Health, and Technology, Faculty of Behavioural, Management, and Social Sciences, University of Twente, Enschede, Netherlands; ^4^Department of Clinical Psychology, Faculty of Social Sciences, Utrecht University, Utrecht, Netherlands

**Keywords:** prolonged grief, PTSD, depression, latent classes, meaning making

## Abstract

**Background:**

The loss of a significant other can lead to variety of responses, including prolonged grief disorder (PGD), posttraumatic stress disorder (PTSD), and depression. The aim of this study was to replicate and extend previous research that indicated that three subgroups of bereaved individuals can be distinguished based one similar post-loss symptom profiles using latent class analysis (LCA). The second aim was to examine whether sociodemographic and loss-related characteristics as well as the extent of meaning making were related to classes with more pervasive psychopathology.

**Methods:**

Telephone-based interviews with 433 Dutch and German speaking persons who had lost a significant other at last 6 months earlier were conducted. Self-rated PGD, PTSD, and depression symptoms were assessed. LCA was conducted and correlates of class-membership were examined using the 3step approach.

**Results:**

The LCA resulted in three distinct classes: a no symptoms class (47%), a moderate PGD, low depression/PTSD class (32%), and a high PGD, moderate depression/PTSD class (21%). A multivariate analysis indicated that female gender, a shorter time since loss, an unexpected loss and less meaning made to a loss were significantly associated with membership to the moderate PGD, low depression/PTSD and high PGD, moderate depression/PTSD class compared to membership to the no symptom class. Losing a child or spouse, a shorter time since loss, and having made less meaning to the loss further distinguished between the high PGD, moderate depression/PTSD symptom class and the moderate PGD, low depression/PTSD class.

**Discussion:**

We found that the majority of individuals coped well in response to their loss since the no symptom class was the largest class. Post-loss symptoms could be categorized into classes marked by different intensity of symptoms, rather than qualitatively different symptom patterns. The findings indicate that perceiving the loss as more unexpected, finding less meaning in the loss, and loss-related factors, such as the recentness of a loss and the loss of a partner or child, were related to class membership more consistently than sociodemographic factors.

## Introduction

In the face of the death of a significant other, people react differently toward their loss. Grief can take up many forms, often including yearning, sadness, and difficulties experiencing positive emotions. The majority of those who experienced a loss resume daily routines and retake part in social or occupational activities within a couple of months and adjust well (1). For others, adjustment is more difficult and grief reactions may take up the form of prolonged grief disorder (PGD), depression, and/ or, in the face of a loss due to traumatic circumstances, posttraumatic stress disorder (PTSD) (2–5).

Factors accounting for maladjustment to loss are manifold. Sociodemographic factors, such as female gender or lower level of education, play a role in the development of PGD, depression and PTSD, while factors inherent to the death and the deceased such as a close kinship to the deceased, and a shorter time since the death were more consistently shown to be associated with PGD (6–8). A violent or sudden nature of the loss has been demonstrated to be associated with PGD, PTSD and depression (9, 10). The way a bereaved person cognitively processes a loss can have an enormous impact on their adjustment. Meaning-making refers to the capacity of an individual to integrate the loss into their belief system about the world and themselves, and to find an explanation or even growth in the loss (11, 12). It has repeatedly been shown that a greater extent of meaning-making is associated with better adjustment to loss as evidenced in lower rates of PGD, depression and PTSD (11, 13–15). Although these cognitive factors have the potential to be targeted in treatment, they are less often investigated (6).

After decades of research and considerable debate (3, 16, 17), PGD was introduced as diagnostic entity in the 11th version of the International Classification of Diseases (ICD-11), which has come into effect on January 1st, 2022. ICD-11 PGD is characterized by separation distress defined as longing or persistent preoccupation with the deceased accompanied by intense emotional pain (e.g., sadness, guilt, anger, or difficulty accepting the death) (18). A diagnosis can be made when the above-mentioned symptoms last for more than 6 months and exceed the social, cultural, or religious norms of the individual's culture or context. While the related concept “Persistent Complex Bereavement Disorder” (PCBD) was included only as condition for further study (section III) within the Fifth Edition of the Diagnostic and Statistical Manual of Mental Disorders (DSM-5), the American Psychiatric Association added prolonged grief disorder in section II in its newest DSM-5-TR edition published in March 2022 (19, 20). PGD shares several features with PTSD and depression and has been shown to be often comorbid, particularly in the wake of violent losses (6, 21). Yet, evidence similarly exists that PGD constitutes a specific syndrome, with separation distress representing a unique feature that is not captured by other disorders (22–25).

To diverge from the notion of examining how disorders do or do not overlap using diagnostic algorithms, some researchers have explored how symptoms of PGD and other indicators of mental health co-occur in bereaved individuals using latent class analysis (LCA). Latent class analysis is a person-centered statistical approach that finds subtypes of related cases in empirical data and thus explores whether there are subgroups of individuals that endorse similar symptom profiles (26). The majority of previous research on PGD using LCA included only two mental health indicators (e.g., PGD and depression or PGD and PTSD) (27, 28). Only a few studies exploring latent classes of PGD and other indicators of mental health included all three mental health indicators PGD, PTSD, and depression. Most LCA studies found three classes: a no symptom class marked by low probabilities to endorse symptoms, a high distress class marked by high probabilities to endorse all PGD, PTSD, and/or depression symptoms, and a PGD class marked by high probabilities to endorse symptoms specific to PGD (29–31).

Moreover, previous studies on LCA mostly relied on the DSM-definitions of the examined disorders. However, definitions of PGD/PCBD and PTSD in ICD-11 vs. DSM-5 differ from each other with regard to the number and content of the symptoms (18, 32). Research has shown that the overlap between the respective definitions is not optimal and that prevalence rates were higher for ICD-11-PGD than for DSM-5 PCBD (33–36). Findings obtained with one definition of a disorder may thus not necessarily apply to another definition of the disorder.

In the light of the ICD-11 coming into effect, aim of this study was to replicate and extend previous findings by examining latent classes of PGD, PTSD, and depression symptoms in bereaved people using the ICD-11 definitions of the disorders. Based on prior LCA studies in bereaved people, we expected to identify at least three latent classes: a (1) low symptom, (2) PGD only, and (3) high symptom class (29–31). Our second aim was to examine correlates of class-membership. We expected that people in the classes with more pervasive symptomatology were more likely to be female, less educated, to make less meaning of their loss, and more likely to have experienced an unexpected or violent loss (29, 31).

## Method Section

### Procedure

This cross-sectional study is part of a longitudinal study on TGI-CA Assessment after Loss in Europe (TALE project), which focuses on the development and validation of an instrument to assess a grief disorder as defined in ICD-11 and DSM-5(-TR) (18, 20). For more information about the study and measures used see https://osf.io/a6hmc/. The study is a joint project at the University of Twente, Groningen, Utrecht, and the Freie University Berlin. It was approved by the Ethical Committee of Psychology of the University of Groningen and the Freie University in Berlin. Data were collected between November 2019 and September 2020 through structured telephone interviews. The breakout of the COVID-19 pandemic occurred after interviews with about 300 participants had already been conducted. We continued our efforts to assess data nonetheless. Pandemic-related restrictions may thus have an effect on part of our data. Interviews were conducted by Dutch and German psychologists (all B.A.) who had received a training on the phenomenology and theoretical background of PGD, the use of questionnaire measures, and interview techniques. This study was pre-registered on the Open Science Framework (see https://archive.org/details/osf-registrations-6hzxw-v1).

### Participants

Participants were recruited through a convenience sampling approach with the help of self-help organizations, mourning cafés, and hospices. In addition, advertisements were placed on social media as well as in the student pool of the respective universities to recruit participants. Interested participants signed up for the study online and provided their informed consent. They were then contacted by the interviewer to schedule a date for the interview. Participants did not receive financial compensation; however, first year psychology students received course credits for taking part in the study. Inclusion criteria required participants to be 18 years or older and to have lost a significant other (i.e., spouse, family member, or friend) at least 6 months prior to the interview. Exclusion criteria were the presence of a psychotic disorder and acute suicidal ideation assessed with single items in the interview. Interviews took about 45 min to complete. A total of 448 participants were recruited (*n* = 221 Dutch speaking, *n* = 227 German speaking). Interviews were not completed with six participants because they fulfilled exclusion criteria. Moreover, nine people were excluded from data analysis as their most significant loss had occurred < 6 months ago, resulting in a final sample size of *N* = 433.

### Measures

The following background and loss-related characteristics were assessed: gender, age of participant, educational level, number of losses, kinship to the deceased, time since loss, cause of loss (i.e., physical illness, accident, suicide, murder/manslaughter, other), unexpectedness of loss (1-5; 1 = totally not unexpected, 5 = completely unexpected), meaning made to the loss [i.e., “To what extent would you say that you were able to give meaning to your loss?”(11) (1 = no meaning through 4 = a good deal of meaning)], history of general psychological support (i.e., “Did you ever receive support for your own problems prior to the death of your loved one from a psychologist, therapist or psychiatrist?” 0 = no, 1 = yes), and received professional bereavement care (i.e., “Did you ever receive support from a psychologist, therapist or psychiatrist related to the death of your loved one?,” 0 = no, 1 = yes).

#### Traumatic Grief Assessment—Clinician Administered

PGD symptoms were assesses using the TGI-CA. The 22-item TGI-CA was developed in the context of the TALE project and is based on 22-item Traumatic Grief Inventory-Self Report Plus (TGI-SR+; 37). The English, German, and Dutch translation of the TGI-CA are freely available *via* the Open Science Framework (https://osf.io/a6hmc/). The TGI-SR+ is a reliable and valid survey to assess PGD symptoms in terms of ICD-11 and DSM-5-TR (37). The TGI-CA deviates from the TGI-SR+ in two aspects: (1) in the TGI-SR+ items were phrased as statement, while in the TGI-CA items were phrased as questions, and (2) in the items and instruction of the TGI-SR+ we refer to “deceased loved one,” while in the TGI-CA we replaced this wording with the first name (e.g., “Albert,” “Mary”) or relationship (e.g., “your husband”) of the deceased person. Participants who reported more than one loss were asked to specify which loss was most distressing or most often in their mind and to relate their answers on the TGA-CA to that loss. Participants rated how often they experienced each symptom during the past month with 1 = never, 2 = seldom, 3 = sometimes, 4 = often, 5 = always. PGD according to ICD-11 criteria is measured using the 12 items that correspond to the ICD-11 classification, namely TGI-CA items 1, 2, 3, 5, 8, 9, 10, 16, 19, 20, 21, 22. Internal reliability in the current study was high (α = 0.90).

#### PTSD Checklist for DSM-5

PTSD symptoms were measured with the Dutch and German version of the PCL-5, a 20-item self-report screening instrument that corresponds to the DSM-5 symptoms of PTSD (38–40). Items are rated on a five-point Likert scale from 1 (not at all) to 5 (extremely). In accordance with previous research (34, 41), six items approximating the ICD-11 operationalization of PTSD were selected to tap ICD-11 PTSD. These items included item 2 (repeated, disturbing dreams), 3 (feeling or acting as if the experience were happening again) 6, (avoidance of internal reminders), 7 (avoidance of external reminders), 17 (being “superalert,” watchful or on guard) and 18 (feeling jumpy, easily startled). Cronbach's alpha levels in the current study was 0.68.

#### Patient-Health-Questionnaire-9

Depression was assessed using the Dutch and German versions of the Patient Health Questionnaire [PHQ-9; (42–44)]. The PHQ-9 is a dimensional screening instrument consisting of nine items based on the diagnostic criteria of depression according to DSM-5. Participants are asked to indicate the severity on a four-point Likert Scale from 1 (not at all) to 4 (almost every day). Internal consistency for the PHQ was 0.80.

#### Work and Social Adjustment Scale (WSAS)

The 5-item Work and Social Adjustment Scale (WSAS) was used to measure functional impairment (45–47). People rated on 9-point scales with anchors 1 = not at all through 9 = severely to what extent the death of their loved one impaired them in their (i) work, (ii) household chores, (iii) social activities, (iv) leisure activities, and (v) close relationships. We added the answer option “not applicable” to the item referring to work. The WSAS demonstrated good internal consistency (α = 0.80).

### Statistical Analysis

Dichotomized item scores of PGD, PTSD, and depression were used as indicators in the LCA. Following prior research (48, 49), the five-point Likert scale of the ICD-11 PGD items of the TGI-CA and ICD-11 PTSD items of the PCL-5 were dichotomized by treating a score of 1 and 2 as symptom absence and a score of 3, 4, and 5 as symptom presence. For depression the four-point Likert scale was recoded by considering a score of 1 and 2 as symptom absent and a score of 3 and 4 as symptom endorsed (48).

The fit of a 1-class through 6-class model was compared using statistical and non-statistical criteria. Model preference relied on a lower (Sample-Size Adjusted) Bayesian Information Criterion (SA-BIC and BIC) and Akaike's Information Criterion (AIC), bootstrap likelihood ratio test (BLRt) with a *p*-value of <0.05, (3) higher entropy R^2^ value, (4) not too small class sample sizes, and (5) accordance with prior LCA research. In case statistical fit indices were indecisive, we relied on the BIC (50). When interpreting LCA symptom profiles, we considered a symptom presentation probability of <0.15 as low, a symptom presentation probability of ≥0.15 and ≤ 0.59 as moderate, and symptom presentation probability of ≥0.60 as high (27). The statistical program LatentGold was used for the LCA (51).

For descriptive purposes we included the total scores on PGD, PTSD, depression, and functional impairment as separate correlates in the model to examine to what extent the classes differed in terms of these severity levels. We did so by using the 3step-approach in LatentGold, which takes the classification error into account when examining correlates of class-membership. We calculated 95% confidence intervals (95% CIs) for class-comparisons. When zero was not included in the 95% CIs the class-comparisons were considered significant.

Correlates of class-membership were examined using again the 3step approach. The following correlates were included simultaneously in a multinomial logistic regression analysis: gender (0 = male, 1 = female), age (in years), educational level (0 = primary, high school, vocational school, 1= university), number of losses (0 = 1 loss, 1 = multiple losses), kinship to the deceased (0 = other than child/spouse, 1 = child/spouse), time since loss (in years), cause of loss (0 = natural, 1 = unnatural), unexpectedness of loss (1-5; 1 = totally not unexpected, 5 = completely unexpected), meaning made to the loss (1 = no sense through 4 = a lot of sense), history of general psychological support (0 = no, 1 = yes), and received professional bereavement care (0 = no, 1 = yes). Based on Chi-square tests and correlation analyses, there was no concern for multicollinearity. Maximum of five responses (1.2%) were missing on the indicators. These missing data were handled using full information maximum likelihood estimation. Missing data on the correlates were handled using listwise deletion. A maximum of one response was missing per correlate.

## Results

### Participants

Table 1 displays the sample characteristics. The sample consisted of 352 female (81.3%) and 81 male participants (18.7%). The mean age was 43 years (SD = 16.89; range: 18-86). The majority of participants indicated having been born in either Germany (*n* = 214, 49.9%) or the Netherlands (*n* = 192; 44.5%). About half of the participants had a university degree as their highest educational attainment (*n* = 216; 49.9%) and about both a quarter indicated high school (*n* = 113; 26.1%) or a vocational education (*n* = 101, 23.3%) as their highest educational attainment.

**Table 1:** Sociodemographic and loss-related characteristics (*N* = 433).

**Gender**				
	Female	*n*, %	352	81.3%
	Male	*n*, %	81	18.7%
Age		M, SD	43.1	16.9
**Education**				
	Primary school	*n*, %	3	0.7%
	High school	*n*, %	113	26.1%
	Vocational Education	*n*, %	101	23.3%
	University	*n*, %	216	49.9%
Multiple loss (yes)		*n*, %	234	54,2%
**Kinship to most significant loss**				
	Partner	*n*, %	119	27.5%
	Child	*n*, %	51	11.8%
	Parent	*n*, %	130	30.0%
	Sibling	*n*, %	16	3.7%
	Grandparent	*n*, %	73	16.9%
	Friend	*n*, %	19	4.4%
	Other	*n*, %	25	5.8%
**Cause of death**				
	Natural loss (e.g., illness, old age)	*n*, %	334	77.1%
	Unnatural loss (e.g., suicide, accident)	*n*, %	99	22.9%
**Expectedness of loss**				
	Totally not unexpected	*n*, %	106	24.5%
	A bit unexpected	*n*, %	72	16.6%
	Quite unexpected	*n*, %	44	10.2%
	Very unexpected	*n*, %	63	14.5%
	Completely unexpected	n, %	147	33.9%
**Meaning made to the loss**				
	No sense	*n*, %	130	30.0%
	A little sense	*n*, %	60	13.9%
	Quite a bit of sense	*n*, %	108	24.9%
	A lot of sense	*n*, %	135	31.2%
Time since loss in months		M, SD	80.4	98.0

*M, mean; SD, standard deviation*.

### Loss-Related Variables

More than half of the participants reported having experienced multiple losses. When asked whose loss was the most difficult to cope with, *n* = 130 (30.0%) reported the loss of a parent, *n* = 119 (27.5%) the loss of their partner, *n* = 73 (16.9%) the loss of a grandparent and *n* = 51 (11.8%) the loss of their child. The average time since the most significant loss was M = 6.7 years (SD = 8.2, range = 6 months-60.7 years). About 20% of the participants had lost their significant other to violent causes and half of the participants indicated that the death of their significant other came “very” or “completely” unexpected. Moreover, 43.9% reported that they had made no or little sense to their loss.

### Latent Class Model Fit

The fit indices for the one through six class models are shown in Table 2. When increasing the number of classes, the AIC and SA-BIC values kept decreasing and all entropy R^2^ values were acceptable (>0.80). The BLRt showed that the two class model showed a significantly better fit than the 1 class model. All other BLRt *p*-values were >0.05. The BIC value was lowest for the three class model. The three class model showed symptom patterns that accords with prior LCA research in bereaved people (29, 52). We therefore selected the three class model as optimal solution.

**Table 2:** Fit indices for the latent class models.

	**LL**	**BIC (LL)**	**AIC (LL)**	**SABIC (LL)**	**Entropy R^**2**^**	**BLRt p-value**	**Class sizes**
1 class model	−5563.47	11290.84	11180.93	11205.16			433
2 class model	−4746.19	9826.27	9602.38	9651.73	0.92	0.054	296/137
3 class model	−4603.75	9711.37	9373.5	9447.98	0.84	0.160	204/139/90
4 class model	−4521.14	9716.12	9264.27	9363.87	0.84	0.236	211/95/66/61
5 class model	−4468.08	9779.99	9214.16	9338.88	0.84	0.268	186/104/54/46/43
6 class model	−4423.76	9861.33	9181.52	9331.37	0.83	0.348	154/108/52/42/41/36

*AIC, Akaike Information Criterion; BIC, Bayesian Information Criterion; BLRt, Bootstrapped Likelihood Ratio test; LL = Loglikelihood; SA-BIC, sample size adjusted Bayesian Information Criterion*.

### Latent Classes of PGD, PTSD, and Depression

**Figure 1:**
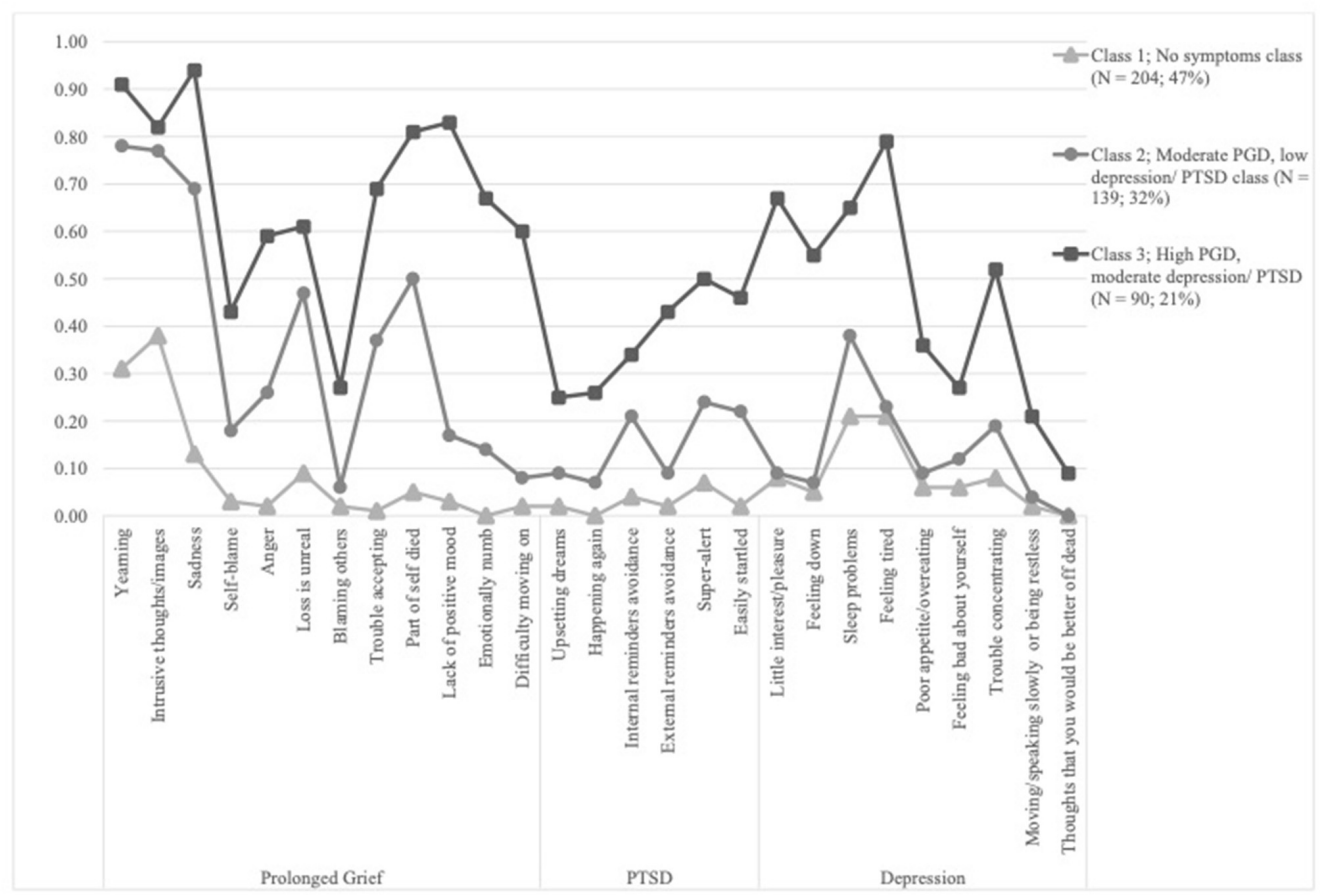
Probability estimates of the three class solution (*N* = 433).

See Figure 1 for probability estimates of the three class model. Figures for other latent class models are displayed in the Supplementary Material. The largest class consisted of 204 individuals (47%) and was characterized by low probability of endorsement of PGD, PTSD, and depression symptoms, except for two PGD symptoms and two depression symptoms that had moderate probability. We labeled this class the “no symptom class.” The second class included 139 people (32%) and was marked by moderate to high probability of endorsement of PGD symptoms and low to moderate probability of endorsement of PTSD and depression symptoms. This class was named “Moderate PGD, low depression/ PTSD class.” The third and smallest class comprised 90 people (21%) that had high probability of endorsement of 8 out of 12 PGD symptoms and moderate probability of all PTSD symptoms and moderate probability for five out of nine depression symptoms. We labeled this class the “High PGD, moderate depression/ PTSD class.” The probability estimates and standard errors are shown in Supplementary Table 1.

The three classes differed significantly in severity levels of PGD, PTSD, depression, and functional impairment, such that the “no symptom class” < “moderate PGD, low depression/ PTSD class” < “high PGD, moderate depression/ PTSD class” (see Table 3). See Supplementary Table 2 for estimates and 95% CIs.

**Table 3:** Univariate associations between PGD, PTSD, depression, and functional impairment levels and classes (*N* = 433).

	**Total sample**	**No symptom class *n* = 204 (47%)**	**Moderate PGD, low depression/PTSD class *n* = 139 (32%)**	**High PGD, moderate depression/PTSD class *n* = 90 (21%)**	**Pairwise comparisons (Class)**
PGD levels, M (SD)	23.54 (9.02)	16.37 (2.86)	25.78 (4.76)	36.32 (7.07)	1 <2 <3
PTSD levels, M (SD)	9.28 (3.35)	7.25 (1.53)	9.71 (2.40)	13.21 (3.84)	1 = 2 <3
Depression levels, M (SD)	15.53 (4.57)	13.32 (3.18)	14.86 (3.07)	21.56 (3.90)	1 <2 <3
Functional impairment levels, M (SD)	17.71 (8.87)	13.57 (7.20)	18.92 (7.86)	25.20 (8.33)	1 <2 <3

*PGD, Prolonged Grief Disorder; PTSD, Posttraumatic Stress Disorder*.

### Background and Loss-Related Correlates of Classes

Correlates of classes were entered simultaneously into the model. Results are displayed in Table 4. Compared to the “no symptom class,” membership to the “high PGD, moderate depression/ PTSD class” was associated with female gender, the loss of a child or spouse, less time since the loss occurred, unexpectedness of the loss, and less meaning made to the loss. Equally relative to the no symptom class, membership to the moderate PGD, low depression/PTSD class was more likely for female participants, those whose loss had occurred more recently, those who experienced their loss as more unexpected and those who had made less meaning of their loss. Moreover, members of the moderate PGD, low depression/PTSD class were more likely to have received bereavement care than members of the no symptoms class. Lastly, compared to the moderate PGD, low depression/PTSD class, membership to the high PGD, moderate depression/PTSD class was associated with the loss of a child or spouse, less time since the loss, and less meaning made to the loss. Estimates and 95% CIs are presented in Supplementary Table 2.

**Table 4:** Correlates of class membership in multivariate model.

	**No symptom class *n* = 204 (47%)**	**Moderate PGD, low depression/ PTSD class *n* = 139 (32%)**	**High PGD, moderate depression/PTSD n = 90 (21%)**	**Pairwise comparisons (Class)**
Gender (1 = Female), *N* (%)	155 (76.0)	119 (85.6)	78 (86.7)	1 <2 =3
Age (in years), M (SD)	42.39 (17.60)	42.35 (16.03)	45.66 (16.49)	1 = 2 = 3
Educational Level (1 = University), *N* (%)	105 (51.5)	74 (53.2)	37 (41.1)	1 = 2 = 3
Kinship to the Deceased (1 = Child/Spouse), *N* (%)	62 (30.4)	53 (38.1)	55 (61.1)	1 = 2 <3
Cause of Loss (1 = unnatural), *N* (%)	36 (17.6)	32 (23.0)	31 (34.4)	1 = 2 = 3
Number of Losses (1 = multiple losses), *N* (%)	114 (56.2)	70 (50.2)	50 (55.6)	1 = 2 = 3
Time Since Loss (in years), M (SD)	8.41 (8.94)	4.39 (5.57)	4.19 (8.63)	1 > 2 > 3
Unexpectedness of Loss (1-5; 1 = totally expected, 5 = completely Unexpected), M (SD)	2.82 (1.61)	3.29 (1.60)	3.78 (1.49)	1 <2 = 3
Meaning Made to the Loss (no meaning through 4 = a good deal of meaning), M (SD)	2.93 (1.14)	2.49 (1.18)	1.90 (1.11)	1 <2 <3
History of general psychological Support (1 = yes), *N* (%)	85 (41.7)	66 (47.5)	44 (48.9)	1 = 2 = 3
Received professional bereavement care (1 = yes), *N* (%)	67 (32.8)	71 (51.1)	50 (55.6)	1 <2, 1 = 3; 2 = 3

## Discussion

This study examined latent classes of PGD, PTSD, and depression symptoms in a sample of Dutch and German bereaved individuals using the ICD-11 definitions of the disorders. The findings of the current study were broadly in line with previous LCA findings using the DSM definitions of the disorders regarding the number of extracted classes and the factors associated with classes with more pervasive psychopathology.

The LCA revealed that a three-class solution fitted the data best. The largest class was termed no symptoms class and comprised almost half of the participants. It was characterized by low item probabilities for almost all PGD, PTSD, and depression symptoms. The moderate PGD, low depression/PTSD class included a third of the participants and was characterized by low to moderate item probabilities for the PTSD and depression symptoms and moderate to high item probabilities for the PGD symptoms. The smallest class, labeled as high PGD, moderate depression/PTSD class, included the remaining 21% of the sample and was marked by high item probabilities for the majority of PGD and three of the depression symptoms and moderate item probabilities for the PTSD and remaining depression symptoms. A three-class solution is consistent with the majority of LCA studies including PGD, PTSD, and depression symptoms (29, 52, 53). However, while previous LCA studies on PGD found classes that were separable by both severity and quality (or “type”) of symptoms (30, 53), classes in the present study differed merely by the severity of symptoms. This indicates that in the current sample, there was no particular PGD response, but rather a high comorbidity of PGD with PTSD and depression within the more symptomatic classes. Members in the high PGD, moderate depression/PTSD class, had, among other symptoms, high probabilities to experience the PGD symptoms “sadness,” “lack of positive mood,” and depression symptoms “little interest/pleasure” and “feeling tired.” These symptoms overlap in content and may thus contribute to higher comorbidity. On the other hand, items indicative of negative sense of self-worth or blame (i.e., items “blame,” “self-blame,” or “feeling bad about oneself”) had low probabilities across all classes. These observations might support previous research that symptoms centering around specific themes are connected across syndromes (49). While some LCA studies found a particular PTSD class (27, 54) it seems plausible that PTSD classes rather emerge in studies with survivors who have been confronted with both loss and trauma in the context of war or forced displacement.

We also tested the differences in PGD, PTSD, functional impairment and depression scores across the classes. The average severity of PGD, PTSD, depression and functional impairment was highest in the high PGD, moderate depression/PTSD class, followed by the moderate PGD, low depression/PTSD class and was lowest in the no symptom class. Only the average PTSD scores did not differ significantly between no symptom and moderate PGD, low depression/ PTSD classes. We thus concluded that the classes were distinguishable meaningfully.

The second aim of this study was to examine the relationship of several sociodemographic and loss-related factors with class membership. In this study, the subjective perception of the loss (i.e., perceived expectedness and meaning made to the loss) and loss-related factors (such as time since loss and relationship to the deceased) predicted class membership more consistently than sociodemographic factors (such as age, gender, education). More specifically, the extent to which participants had made meaning of their loss and the recentness of the loss distinguished between all classes and having lost a child or spouse additionally distinguished between the moderate PGD, low depression/PTSD and high PGD, moderate depression/PTSD class, while age and education were found to be unrelated. Our finding that the extent of meaning made to the loss clearly distinguishes between the classes is in line with previous findings that showed that less meaning made to a loss was associated with more PGD symptoms (55). This has important implications for clinical practice. Compared to sociodemographic or loss-related factors that are invariant (e.g., gender, relationship to the deceased) or systemic in nature (e.g., time since loss), reconstructing meaning can be addressed in treatment and thus facilitate adjustment. A meaning reconstruction approach through writing assignments and a ritual of remembrance may be a promising intervention to finding meaning and reducing PGD symptoms (56, 57). However, future research should also investigate whether meaning making is indeed a constructive coping strategy that results in “actual” meaning-finding or rather the result of a cognitive bias that also contains illusory aspects (58).

In addition to recentness of the loss and meaning making, having perceived the loss as unexpected, having received professional grief support, and female gender distinguished between the no symptom and the moderate PGD, low depression/PTSD class. Contrary to our hypothesis, the cause of the loss was unrelated to class membership when taking other covariates into account. Our findings suggest that in the current sample, it was rather the subjective perception of the loss (expected vs. unexpected) than the objective cause (violent vs. non-violent loss) that was associated with adverse mental health outcomes. Cause and unexpectedness of death are related constructs as most violent deaths are unexpected and it might be that in our sample, cause did not explain unique variance beyond the unexpectedness of a loss. This accords also with prior findings that an objective measure of unexpectedness (measured as “number of days between forewarning of death and the actual death”) was not associated with PGD symptoms (59), while a subjective measure of perceived unexpectedness was linked to elevated levels of PGD symptoms (60, 61). Further recent research demonstrates that bereaved persons who experienced their loss as unexpected reported higher levels of PGD, even when other variables were controlled (62, 63).

It is a promising finding that among the more symptomatic classes, more than half had sought professional grief support, potentially indicating positive attitudes toward professional help within the current sample. However, half of them did not seek grief support which points again to a treatment gap in bereavement care which has also been identified in prior research (64). It seems conceivable that individuals who had developed symptoms after their loss were also more likely to seek professional grief support. There is some evidence highlighting potential barriers to seeking mental health care among bereaved people, such as thinking the problems will naturally disappear, pain of talking about the loss, and difficulty finding help (64, 65). These two prior studies on barriers to seek support were conducted in people bereaved by traffic accidents and parents who lost a child due to cancer. It would be desirable for future research to examine these barriers in people bereaved by other causes. Finding out about these barriers to professional grief support may entail important knowledge how to approach individuals in need of mental health care, particularly in light of the inclusion of PGD in the ICD-11, which enables evidence-based treatment covered by health insurance.

### Limitations

Some aspects should be discussed that may impact the interpretation of results. Data for the current study were gathered before and during the breakout of the COVID-19 pandemic and the pandemic-related restrictions and additional stressors are likely to have had an impact on the well-being of our participants. Research to date has focused primarily on the negative consequences of the pandemic for adults who have suffered a loss due to the virus or during the pandemic (66, 67). Less is known about the impact of the pandemic on the grief intensity of people who have experienced a loss before the pandemic (68). It is possible that social isolation and the respective lack of social support, increased worries about oneself and relatives have increased the mental health burden. For others, the pandemic may also have had a positive effect by providing more time to process a loss. It would be desirable if future research addressed the specific impact of the pandemic on the grief process of the bereaved.

A few limitations should be considered when interpreting the results. Data for the current study were gathered in telephone-based interviews. Even though interviewers were trained, it is possible that differences in data assessment between telephone and in-person administration exist. For example, telephone-based interviews may enable participants to talk more openly about their distress, but misunderstandings due to the lack of transmission of non-verbal cues are possible. Second, we used a selection of PCL-5 items to assess ICD-11 PTSD. Although the PCL-5 is a validated instrument to assess PTSD for DSM-5, it was not developed to assess ICD-11 PTSD. However, items in the PCL-5 are mostly congruent and equivalent in content with the formulation of items within the International Trauma Questionnaire, an instrument specifically developed for the assessment of ICD-11 PTSD (69). Third, different guidelines for interpreting probability estimates have been suggested (27, 70). For the purpose of comparability, we used cut-off scores that are most commonly used in the field of latent classes of PGD. These interpretation guidelines have a relatively low threshold to consider the probability of a symptom as “moderate” or “high.” Similarly, we followed other LCA research (28, 29) by using the three highest answer options on the TGI-CA as symptom presence. For the aforementioned reasons, a possible risk of pathologizing scores should be considered. Fourth, expectedness of loss and meaning making were assessed with one item, respectively. Future research could use multi-item or more observational instruments to assess these constructs, for example the *Grief and Meaning Reconstruction Inventory* (71). Fifth, despite our efforts to include male participants in our study, female participants predominate this sample by far. Results can thus not be generalized to a male sample. Some evidence suggests that male forms of grief may be different, e.g., that socially constructed ideals may encourage stoic behavior or expression of grief as anger (72, 73). Even though it seems challenging, future research should increase efforts to include men in grief-research to a gender-balanced level. Last, the cross-sectional nature of this study does not allow to draw conclusions about the causal relationship between variables.

## Conclusion

In conclusion, LCA revealed three subgroups differing in symptom severity of PGD, PTSD, and depression in a large sample of Dutch and German bereaved individuals. While the majority of bereaved individuals coped well in response to their loss, results show that women, those who had lost a close relative recently and unexpectedly and those who expressed difficulties to make meaning of their loss had a higher probability to show psychological symptoms, in particular PGD and depression.

In cases of more pervasive psychopathology, addressing meaning reconstruction in treatment might be an important pathway to help bereaved individuals to integrate the loss into their world view. More than half of participants in the more pervasive symptom classes received professional grief support, which can be interpreted as an encouraging indication of the openness toward professional support among those in need of help.

## Data Availability Statement

The datasets presented in this study can be found in online repositories. The names of the repository/repositories and accession number(s) can be found at: https://osf.io/a6hmc/.

## Ethics Statement

The studies involving human participants were reviewed and approved by Freie University Berlin and University of Groningen. The patients/participants provided their written informed consent to participate in this study.

## Author Contributions

CH and LL are responsible for the study design and concept. Data were assessed by CH, MF, HH, and LL. LL undertook the statistical analyses. CH and LL drafted the manuscript, which was critically revised by CK, HH, and MF. All authors contributed to and have approved the final manuscript.

## Conflict of Interest

The authors declare that the research was conducted in the absence of any commercial or financial relationships that could be construed as a potential conflict of interest.

## Publisher's Note

All claims expressed in this article are solely those of the authors and do not necessarily represent those of their affiliated organizations, or those of the publisher, the editors and the reviewers. Any product that may be evaluated in this article, or claim that may be made by its manufacturer, is not guaranteed or endorsed by the publisher.
