# Supplementary material for: A Latent Class Analysis on Symptoms of Prolonged Grief, Post-Traumatic Stress, and Depression Following the Loss of a Loved One
Source: Front Psychiatry. 2022 May 27;13:878773. doi: 10.3389/fpsyt.2022.878773 (PMC9184516; doi:10.3389/fpsyt.2022.878773)
Supplement: Supplementary file 1 [file Table_1.DOCX]

**Supplementary Material**

*Supplementary Table 1*. Probability estimates of three class model (N = 433)

|  |  | Class 1; No symptom class (N = 204; 47%) | | Class 2; moderate PGD, low depression/ PTSD class (N = 139; 32%) |  | Class 3; high PGD, moderate depression/ PTSD (N = 90; 21%) |  |
| --- | --- | --- | --- | --- | --- | --- | --- |
|  |  | B | SE | B | SE | B | SE |
| Prolonged grief | |  |  |  |  |  |  |
|  | Yearning | 0.31 | 0.04 | 0.78 | 0.04 | 0.91 | 0.03 |
|  | Intrusive thoughts/images | 0.38 | 0.04 | 0.77 | 0.04 | 0.82 | 0.04 |
|  | Sadness | 0.13 | 0.03 | 0.69 | 0.05 | 0.94 | 0.03 |
|  | Self-blame | 0.03 | 0.01 | 0.18 | 0.04 | 0.43 | 0.05 |
|  | Anger | 0.02 | 0.01 | 0.26 | 0.05 | 0.59 | 0.05 |
|  | Loss is unreal | 0.09 | 0.02 | 0.47 | 0.05 | 0.61 | 0.05 |
|  | Blaming others | 0.02 | 0.01 | 0.06 | 0.02 | 0.27 | 0.05 |
|  | Trouble accepting | 0.01 | 0.01 | 0.37 | 0.05 | 0.69 | 0.05 |
|  | Part of self died | 0.05 | 0.02 | 0.50 | 0.05 | 0.81 | 0.04 |
|  | Lack of positive mood | 0.03 | 0.01 | 0.17 | 0.04 | 0.83 | 0.05 |
|  | Emotionally numb | 0.00 | 0.00 | 0.14 | 0.03 | 0.67 | 0.05 |
|  | Difficulty moving on | 0.02 | 0.01 | 0.08 | 0.03 | 0.60 | 0.06 |
| Posttraumatic stress disorder | |  |  |  |  |  |  |
|  | Upsetting dreams | 0.02 | 0.01 | 0.09 | 0.03 | 0.25 | 0.05 |
|  | Happening again | 0.00 | 0.00 | 0.07 | 0.02 | 0.26 | 0.05 |
|  | Internal reminders avoidance | 0.04 | 0.02 | 0.21 | 0.04 | 0.34 | 0.05 |
|  | External reminders avoidance | 0.02 | 0.02 | 0.09 | 0.03 | 0.43 | 0.06 |
|  | Super-alert | 0.07 | 0.02 | 0.24 | 0.04 | 0.50 | 0.06 |
|  | Easily startled | 0.02 | 0.01 | 0.22 | 0.04 | 0.46 | 0.06 |
| Depression | |  |  |  |  |  |  |
|  | Little interest/pleasure | 0.08 | 0.02 | 0.09 | 0.03 | 0.67 | 0.06 |
|  | Feeling down | 0.05 | 0.02 | 0.07 | 0.02 | 0.55 | 0.06 |
|  | Sleep problems | 0.21 | 0.03 | 0.38 | 0.05 | 0.65 | 0.05 |
|  | Feeling tired | 0.21 | 0.03 | 0.23 | 0.04 | 0.79 | 0.05 |
|  | Poor appetite/overeating | 0.06 | 0.02 | 0.09 | 0.03 | 0.36 | 0.05 |
|  | Feeling bad about yourself | 0.06 | 0.02 | 0.12 | 0.03 | 0.27 | 0.05 |
|  | Trouble concentrating | 0.08 | 0.02 | 0.19 | 0.04 | 0.52 | 0.05 |
|  | Moving/speaking slowly or being restless | 0.02 | 0.01 | 0.04 | 0.02 | 0.21 | 0.04 |
|  | Thoughts that you would be better off dead | 0.00 | 0.00 | 0.00 | 0.00 | 0.09 | 0.03 |

*Note.* B = probability estimate, SE = Standard error.

.

*Supplementary Table 2.* Correlates of classes

|  | B | SE (B) | 95% confidence interval | |
| --- | --- | --- | --- | --- |
| No symptom class vs. moderate PGD, low depression/ PTSD |  |  |  |  |
| *Univariate analyses* |  |  |  |  |
| Prolonged grief disorder levels | **0.93** | 0.11 | 0.72 | 1.14 |
| Posttraumatic stress disorder levels | **0.81** | 0.11 | 0.60 | 1.02 |
| Depression levels | **0.22** | 0.05 | 0.12 | 0.32 |
| Functional impairment levels | **0.11** | 0.02 | 0.07 | 0.15 |
| *Multivariate analyses* |  |  |  |  |
| Gender (1 = female) | **0.90** | 0.36 | 0.20 | 1.60 |
| Age in years | 0.02 | 0.01 | 0.00 | 0.04 |
| Education (1= university) | -0.05 | 0.29 | -0.62 | 0.53 |
| Kinship (1 = child/spouse) | -0.32 | 0.40 | -1.10 | 0.47 |
| Cause of loss (1 = unnatural) | 0.32 | 0.35 | -0.37 | 1.01 |
| Number of losses (1 = multiple loss) | -0.06 | 0.28 | -0.60 | 0.48 |
| Time since loss (in years) | **-0.09** | 0.04 | -0.16 | -0.02 |
| Expectedness of loss (1-5; 1 = totally not unexpected, 5 = completely unexpected) | **0.25** | 0.10 | 0.06 | 0.44 |
| Meaning made (no meaning through 4 = a good deal of meaning) | **-0.48** | 0.13 | -0.74 | -0.22 |
| General support (1 = yes) | 0.19 | 0.29 | -0.38 | 0.75 |
| Grief support (1 = yes) | **0.70** | 0.31 | 0.09 | 1.31 |
| No symptom class vs. high PGD, moderate depression/ PTSD class |  |  |  |  |
| *Univariate analyses* |  |  |  |  |
| Prolonged grief disorder levels | **1.26** | 0.12 | 1.03 | 1.49 |
| Posttraumatic stress disorder levels | **1.20** | 0.12 | 0.96 | 1.44 |
| Depression levels | **0.72** | 0.08 | 0.56 | 0.88 |
| Functional impairment levels | **0.21** | 0.03 | 0.16 | 0.26 |
| *Multivariate analyses* |  |  |  |  |
| Gender (1 = female) | **1.03** | 0.52 | 0.01 | 2.06 |
| Age in years | 0.02 | 0.01 | 0.00 | 0.04 |
| Education (1= university) | -0.50 | 0.34 | -1.17 | 0.16 |
| Kinship (1 = child/spouse) | **0.90** | 0.43 | 0.06 | 1.74 |
| Cause of loss (1 = unnatural) | 0.48 | 0.40 | -0.29 | 1.26 |
| Number of losses (1 = multiple loss) | 0.07 | 0.33 | -0.57 | 0.72 |
| Time since loss (in years) | **-0.22** | 0.06 | -0.33 | -0.11 |
| Expectedness of loss (1-5; 1 = totally not unexpected, 5 = completely unexpected) | **0.32** | 0.12 | 0.08 | 0.55 |
| Meaning made (no meaning through 4 = a good deal of meaning) | **-0.82** | 0.15 | -1.11 | -0.53 |
| General support (1 = yes) | 0.27 | 0.35 | -0.41 | 0.95 |
| Grief support (1 = yes) | 0.56 | 0.37 | -0.17 | 1.28 |
| Moderate PGD, low depression/ PTSD class vs. high PGD, moderate depression/ PTSD class |  |  |  |  |
| *Univariate analyses* |  |  |  |  |
| Prolonged grief disorder levels | **0.33** | 0.04 | 0.26 | 0.41 |
| Posttraumatic stress disorder levels | **0.39** | 0.06 | 0.27 | 0.51 |
| Depression levels | **0.50** | 0.06 | 0.38 | 0.63 |
| Functional impairment levels | **0.10** | 0.02 | 0.05 | 0.14 |
| *Multivariate analyses* |  |  |  |  |
| Gender (1 = female) | 0.14 | 0.58 | -0.99 | 1.27 |
| Age in years | 0.00 | 0.01 | -0.03 | 0.02 |
| Education (1= university) | -0.45 | 0.33 | -1.10 | 0.19 |
| Kinship (1 = child/spouse) | **1.22** | 0.43 | 0.38 | 2.06 |
| Cause of loss (1 = unnatural) | 0.17 | 0.38 | -0.58 | 0.91 |
| Number of losses (1 = multiple loss) | **0.13** | 0.33 | -0.51 | 0.78 |
| Time since loss (in years) | **-0.13** | 0.06 | -0.25 | -0.01 |
| Expectedness of loss (1-5; 1 = totally not unexpected, 5 = completely unexpected) | 0.06 | 0.12 | -0.17 | 0.30 |
| Meaning made (no meaning through 4 = a good deal of meaning) | **-0.33** | 0.14 | -0.61 | -0.06 |
| General support (1 = yes) | 0.09 | 0.34 | -0.59 | 0.76 |
| Grief support (1 = yes) | -0.14 | 0.35 | -0.83 | 0.55 |

**Supplementary Figure 1.** Probability estimates one class solution (N = 433).

**Supplementary Figure 2.** Probability estimates two class solution (N = 433).

**Supplementary Figure 3.** Probability estimates four class solution (N = 433).

**Supplementary Figure 4.** Probability estimates five class solution (N = 433).

**Supplementary Figure 5.** Probability estimates six class solution (N = 433).
